# Taking methodological pluralism seriously: considerations based on the work of Norbert Groeben

**DOI:** 10.3389/fpsyg.2023.1215737

**Published:** 2023-09-25

**Authors:** Fabian Hutmacher

**Affiliations:** Human-Computer-Media Institute, Julius-Maximilians-University Würzburg, Würzburg, Germany

**Keywords:** methodological pluralism, quantitative methods, qualitative methods, replication crisis, Norbert Groeben

## 1. Introduction

Rumor has it that psychology is in deep crisis. Based on the observation that many psychological studies cannot be replicated (Open Science Collaboration, 2015; Marsman et al., 2017), this crisis has often been termed a *replication crisis* (Simmons et al., 2011; Pashler and Wagenmakers, 2012). Most recommendations for addressing this crisis and for ensuring the quality of psychological science have either focused on improving existing statistical and quantitative-experimental methods or emphasized the importance of embracing open science practices (e.g., through preregistrations as well as sharing data and materials; Nosek et al., 2015). At the same time, however, there are also researchers suggesting that psychology should additionally be more open to embracing cultural diversity (Henrich et al., 2010; Apicella et al., 2020), cross-temporal variation (Hutmacher and Mayrhofer, 2021, 2023; Muthukrishna et al., 2021; Hutmacher, 2022) as well as the insights that the psychological humanities have to offer (Teo, 2017; Malich and Rehmann-Sutter, 2022). Arguably, such a diversified understanding of the subject matter of psychology would also be mirrored in the application of a diversified set of research methods that could account for the complexity and multilayeredness of the phenomena under investigation (e.g., Mayrhofer and Hutmacher, 2020; Hutmacher and Mayrhofer, 2021).

While moving (Western) academic psychology toward more methodological pluralism—in the sense that a greater range of different methods is accepted as legitimate and used for studying psychological phenomena—may sound desirable, it is far from clear what embracing methodological pluralism would mean for everyday research practices. On the one hand, there are researchers arguing in favor of an “anything goes” attitude that allows researchers to use the methods they prefer and asks them to develop tolerance for other researchers subscribing to other methodologies (cf. Zitzmann and Loreth, 2021). On the other hand, it has been pointed out that methodological pluralism should not be equated with such an “anything goes” attitude but has to be based on a thorough analysis of the strengths and weaknesses of the different approaches and paradigms (Yanchar and Slife, 1997, 2000; Healy, 2012). In line with this, Teo (2021) emphasized that methods need to “do justice” to the psychological phenomena under investigation, implying that a particular method may be adequate under certain conditions but not under others. Of course, this leads to the intricate key question as to which method is appropriate for studying which phenomenon. One attempt to answer this question was made by Groeben (1986) in his book *Handeln, Tun, Verhalten als Einheiten einer verstehend-erklärenden Psychologie* (*Acting, Doing, Behaving as Units of an Interpretive-Explanatory Psychology*). Although it is almost 40 years since the book has been published, it may still help to start thinking about what “doing justice” to psychological phenomena would entail.

## 2. How to take methodological pluralism seriously

Groeben distinguishes between two ways of investigating human mind and behavior that can be traced back through the entire history of academic psychology. On the one hand, those who share a natural science perspective on psychology typically approach human behavior using quantitative-experimental methods and aim at establishing covering laws that can explain this behavior. On the other hand, those who hold a perspective grounded in the humanities typically approach human behavior using hermeneutic methods and aim at describing behavior. Groeben criticizes both approaches as being incomplete. The natural science perspective is reductionist as it neglects the fact that humans are not passive objects in a pre-determined universe but rational and autonomous beings who are able to act intentionally and to communicate in meaningful ways. As Groeben sees it, the perspective grounded in the humanities, which puts great emphasis on acknowledging the subjectivity and the freedom of the individual and thus has a richer understanding of human nature, does not offer a viable alternative as it has abandoned the idea of explaining human behavior and is satisfied with merely describing it.

### 2.1. Acting, doing, and behaving as three levels of analysis

In order to overcome this deadlock, Groeben tries to develop a theoretical framework that keeps the strengths and eliminates the weaknesses of both perspectives and that he labels “hermeneutic natural science” (hermeneutische Naturwissenschaft). Groeben suggests distinguishing between three basic levels of analysis: acting (“Handeln”), doing (“Tun”), and behaving (“Verhalten”). *Acting* is intentional, planned, meaningful, and oriented toward goals and norms. When the subjective intention and the objective motivation do not align, that is, in situations, in which humans are not fully aware of their goals and motives, they are *doing* things. When individuals are driven by universal hard-wired mechanisms, they *behave*. Groeben holds that “acting” is located on a higher level of abstraction than “doing,” and that “doing” is located on a higher level of abstraction than “behaving.” Following the idea that theories should be as parsimonious as possible, Groeben argues that researchers should start at the highest level of abstraction (i.e., acting) and that it is only allowed to step down the hierarchy to the levels of doing and behaving when it becomes clear that an analysis at a higher level of abstraction is insufficient.

Moreover, Groeben associates the investigation of each level of analysis with a specific methodology. For the analysis of “acting,” Groeben suggests the so-called dialogue-hermeneutic method. In short, the dialogue-hermeneutic method helps researchers and participants to reconstruct the participants' individual cognitions in a joint effort. In a second step, it is investigated whether these reconstructions derived by the dialogue-hermeneutic method accurately map with observable behavior, that is, whether the identified intentions and goals can be considered to play a causal role in the individual's actions (for details, see Groeben and Scheele, 2000). According to Groeben, combining these two steps ensures that the resulting analysis does not only describe but explain the individual's actions. If the reconstructions do not map with observable behavior, the researcher has encountered a case of “doing,” in response to which the researcher switches to a monolog-hermeneutic procedure. That is, the researcher develops a reconstruction of the things that an individual has *done* that also considers unconscious motives, goals, and intentions. In case researchers are concerned with analyzing instances of “behaving,” hermeneutic methods are skipped; instead, it is analyzed which environmental determinants drive the way an individual *behaves*.

### 2.2. Learning from Groeben's theoretical framework

What can be learned from Groeben's framework for today's debates about methodological pluralism and the idea of “doing justice” to psychological phenomena? First, as Groeben points out, the conflict between those researchers in psychology who hold a natural science perspective and those wo prefer a perspective grounded in the humanities is not a new one but can be traced back to the very beginnings of psychology as an academic discipline. Thus, a theoretical framework that tries to bridge the gap between these two perspectives and that provides an answer to the question as to how methodological pluralism can be put into action seems highly desirable.

Second, and in line with the previous point, Groeben raises awareness for the fact that the choice of research methods should be based on the kind of research question that is being asked—and not vice versa. Importantly, Groeben demonstrates that quantitative-experimental methods may be insufficient when analyzing human behavior as the behavior of rational agents who use language as a medium of communication and of constructing meaning. This seems particularly noteworthy as many psychological researchers still limit themselves to a predetermined selection of quantitative-experimental methods, rather than considering which research method is most appropriate for their specific research question (comparable lines of critique can be found in Jüttemann, 1983; Yanchar et al., 2005; Mayrhofer and Hutmacher, 2020; Lamiell and Slaney, 2021). In other words, Groeben's framework implies that the decision to use a certain method should always be accompanied by practices of reflexivity throughout the entire research process (cf. Jamieson et al., 2023).

Third, as Groeben also notes, the natural science perspective has largely dominated academic psychology throughout the twentieth century. From today's perspective, one may add that it continues to do so in the beginning of the twenty-first century. Emphasizing that humans have the capacity for acting autonomously and rationally and that academic psychology would benefit from shifting more attention to the “acting”-aspects of human behavior can be seen as an important reminder in this context. That is, while Groeben acknowledges that the human mind can be studied and analyzed from a wide range of different perspectives, namely perspectives derived from the natural sciences as well as perspectives derived from the humanities (for similar ideas, see, e.g., Watanabe, 2010), he also emphasizes that the latter kind of perspectives have often been neglected. The idea that it is important to strengthen the role of the psychological humanities has been underlined in recent publications and is as relevant today as it was 40 years ago (cf. Teo, 2017; Malich and Rehmann-Sutter, 2022).

## 3. Discussion

Even though Groeben's theoretical framework provides an interesting attempt to address the question as to how methodological pluralism can be put into action, it comes with several limitations. To begin with, it has been demonstrated that it is notoriously difficult to reconstruct the meaning of an observable behavior (cf. Shweder, 1977), casting doubt on the possibility of determining beyond any doubt whether one is facing an instance of acting, doing, or behaving. In line with this, it appears far from clear that the three levels of analysis are as independent as postulated by Groeben and that their investigation needs to be put into a hierarchical order. Quite the contrary, it seems reasonable to assume that conscious goals and intentions, unconscious motives, and hard-wired behavior tendencies often all contribute to a specific observable behavior. If this were the case, one could still hold that contemporary psychology devotes too little attention to analyzing humans as intentional agents, that all levels need to be taken into account to arrive at an adequate understanding of psychological phenomena, and that researchers should reflect in more detail about the level of analysis at which they are operating. That is, keeping in mind the distinction between acting, doing, and behaving may help researchers to improve the conceptual clarity of their theories and the methodological rigor of their investigations (cf. Bringmann et al., 2022). However, choosing a level of analysis would ultimately be up to the researcher's focus and interest.

Apart from that, academic psychology has developed a host of quantitative and qualitative as well as mixed methods suited for specific purposes (cf. Bryman, 2006; Schoonenboom and Johnson, 2017), which do not fit with the narrow set of methodological categories that Groeben has proposed. Hence, strictly adhering to the scheme proposed by Groeben may ultimately rather endanger than support a productive and methodologically diversified academic psychology. In the worst case, one runs the risk of introducing new methodological norms: That is, taking Groeben's framework as the last word on how researchers can “do justice” to the psychological phenomena under investigation would mean putting a premature end to a debate that is in fact still ongoing. One aspect of this debate that has not been mentioned so far and that also does not play a role in Groeben's framework is the complicated question whether the different perspectives on the human psyche are commensurable in the sense that different methodological approaches associated with these perspectives simply capture different aspects of the same phenomena or whether these different perspectives are linked to ontologically different phenomena (cf. Koch, 1993). In accordance with this, Teo (2021) argued that a method that is “doing justice” to a certain kind of psychological phenomenon may be problematic when being applied to another kind of psychological phenomenon, suggesting that there are limits to the application of each method. Taken together, this demonstrates that implementing a version of methodological pluralism that goes beyond a mere “anything goes” attitude and that “does justice” to the psychological phenomena under investigation is not only desperately needed but also far from trivial to achieve. While Norbert Groeben may ultimately not be able to offer a viable framework, he at least offers a thought-provoking starting point for reflecting upon a version of methodological pluralism that could provide a satisfying answer to psychology's current crises.

## Author contributions

FH developed the research idea and wrote the manuscript.
